# Building the Field of Health Policy and Systems Research: An Agenda for Action

**DOI:** 10.1371/journal.pmed.1001081

**Published:** 2011-08-30

**Authors:** Sara Bennett, Irene Akua Agyepong, Kabir Sheikh, Kara Hanson, Freddie Ssengooba, Lucy Gilson

**Affiliations:** 1 Health Systems Program, Johns Hopkins Bloomberg School of Public Health, Baltimore, Maryland, United States of America; 2 Ghana Health Service, Accra, Ghana and University of Ghana School of Public Health, Legon, Ghana; 3 Public Health Foundation of India, New Delhi, India; 4 Department of Global Health and Development, London School of Hygiene and Tropical Medicine, London, United Kingdom; 5 School of Public Health, Makerere University, Kampala, Uganda; 6 School of Public Health and Family Medicine, University of Cape Town, Cape Town, South Africa

## Abstract

In the final article in a series addressing the current challenges and opportunities for the development of Health Policy and Systems Research (HPSR), Sara Bennett and colleagues lay out an agenda for action moving forward.

***PLoS Medicine* Series on HPSR**

Following the *First Global Symposium on Health Systems Research* in Montreux in November 2010, *PLoS Medicine* commissioned three articles on the state-of-the-art in Health Policy and Systems Research (HPSR). Three Policy Forum articles, authored by a diverse group of global health academics, critically examine the current challenges to the field and lay out what is needed to build capacity in HPSR and support local policy development and health systems strengthening, especially in low and middle income countries.

*Paper 1*. Kabir Sheikh and colleagues. Building the Field of Health Policy and Systems Research: Framing the Questions.

*Paper 2*. Lucy Gilson and colleagues. Building the Field of Health Policy and Systems Research: Social Science Matters

*Paper 3*. Sara Bennett and colleagues. Building the Field of Health Policy and Systems Research: An Agenda for Action

**Summary Points**

- There is an urgent need to build the Health Policy and Systems Research (HPSR) field and in particular to develop understanding across different disciplinary boundaries.
- The development of HPSR is impeded by a cluster of related issues, namely (i) a heavy reliance on international funding for HPSR, (ii) an excessive focus on the direct utility of HPSR findings from specific studies, and (iii) a tendency to under-value contributions to HPSR from social sciences.
- Innovations in funding HPSR are needed so that local actors, including policy-makers, civil society, and researchers, have a greater say in determining the nature of HPSR conducted.
- Strategic investment should be made in promoting a greater shared understanding of theoretical frames and methodological approaches for HPSR including, for example, the development of HPSR journals, methodological workshops, and shared HPSR teaching curricula.
- Dedicated and supportive homes for HPSR need to be found within universities, and also be developed as independent research institutes.

## Introduction

The lack of clarity and shared understanding regarding the scientific foundations of Health Policy and Systems Research (HPSR) [1] potentially has very negative consequences for the field [2]. Disagreement over the value of different types of theoretical frameworks and research methods can lead to inappropriate evaluations of research proposals, contradictory reviews of the same paper, and delays in publication. Excessive time may be spent communicating broad frameworks to other researchers within HPSR, inhibiting progression to more detailed and specific conversations. Communication barriers may discourage inter-disciplinary collaboration, driving researchers back to their disciplinary safety zones, and creating potential for conflict that may discourage younger researchers who may be less secure in their career from staying in the field. As the second paper in this series concluded [1], there is an urgent need to build understanding across disciplinary boundaries. This final paper in the “Building the Field of HPSR” series turns to practical questions concerning how to remove structural barriers that currently inhibit the development of the HPSR field and thus unlock HPSR capacities.

HPSR suffers from many of the same problems as other branches of health research in low- and middle-income countries (LMICs): major imbalances between the resources available in high- versus low- and middle-income contexts [3], acute shortages of skilled researchers (especially senior ones), and relatively few organizations that house HPSR expertise [4]. Historically, low levels of funding for HPSR compared to clinical or biomedical research have compounded these problems. Many papers provide relevant recommendations to address health research capacity issues in LMICs [5]–[8]. However, there is also a nexus of issues specific to HPSR that currently constrains development of the field. This paper builds on the analysis of the previous papers in this series [1],[9] to investigate the practical problems faced and then develops an agenda for building the HPSR field.

## Unpacking the Problem

The development of HPSR is affected by a series of interconnected problems:

- a heavy reliance on international funding for HPSR;
- an excessive focus on the direct utility of HPSR findings from specific studies;
- a tendency to under-value contributions to HPSR from social sciences.

While the first of these problems may not be unique to HSPR, its significance is: HPSR—unlike clinical or biomedical research—should be driven by understanding of local contexts. At all stages of the research endeavor, from prioritization of research questions, to conceptualization and conduct of the research, to interpretation of and communication of findings, HPSR will benefit from being embedded within a particular context and close engagement with local actors.

In LMICs (particularly low-income countries), current funding for HPSR comes predominantly from external sources, notably international and bilateral agencies, but also via sub-contracts through larger research consortia typically led by Northern researchers [10]. A further important source of funding for “HPSR-type” analysis (though rarely undertaken with the rigor of research) comes from short-term consulting contracts. Consultancies commissioned by aid agencies may crowd out HPSR that is responsive to local needs [11].

Global funding for HPSR is frequently focused on programmatic or operational questions with a primary concern of how to expedite the scale up of priority services [10]. It is less likely to address deeper, more structural questions (such as how to promote accountability in health systems) or to support action research that actively engages stakeholders in improving health systems. In part, this is due to the nature of new funding agencies in the field who are frequently focused on achieving global targets, and thus prioritize research that leads to generalizable conclusions supporting decision-making and service scale up, across LMIC contexts. It is unclear to what extent local actors in LMIC health systems would frame their research concerns in the same way as global stakeholders.

One of the strengths of HPSR is that it is frequently of direct instrumental value, leading to changes in policy and practice. But research findings can also be influential in less direct ways, for example, by shifting the framing of health policy debates, and gradually influencing the nature of dialogue [12],[13]. These indirect influences of research can be more significant than direct ones. Frequently, direct use of research addresses marginal changes or technical questions such as “Should immunization services be delivered through campaigns or fixed health centers?” or “What salary increase would incentivize health workers to stay in rural areas?” In contrast, research evidence that shapes understanding of the complexity of a problem may ultimately lead to more substantial reforms and greater impacts—but through longer and more circuitous routes [14]. This kind of game-changing research often challenges assumptions and established ways of working, and for this reason it may be neglected or actively resisted by powerful global and national actors [9]. Further, by narrowing the focus of HSPR to specific, short-term questions, opportunities are missed to engage in long-term blue-sky thinking that may address the health systems challenges of tomorrow.

The belief that HPSR should have direct impacts upon policy has led to an emphasis on the more direct forms of knowledge translation such as the development of policy briefs, and hosting of workshops to disseminate research findings. While such activities are important, there is a danger that the less visible, structural factors that critically influence the policy/research interface and involve, for example, the development of long-term relationships between researchers and policy-makers built upon mutual trust and familiarity with the contexts within which each other works, are neglected [15].

The phenomena identified above also contribute to the third concern, the relative neglect of social science within HPSR. The perspectives of new funders to the HPSR field are informed by the type of research (frequently positivist and biomedical) that they are accustomed to funding as well as by their perception of current priority research questions. Relativist perspectives often provide nuanced insights into how to implement a policy or why a policy is ineffective, but rarely offer discrete interventions as policy solutions. Building on the user fee example from the second paper in this series (Gilson et al. [1]), Table 1 illustrates the nuanced policy lessons that relativist perspectives may provide compared to the more direct policy lessons sometimes offered by positivist investigations.

**Table 1:** Possible policy implications of alternative types of research on user fees.

Perspective	Typical Research Question	Illustrative Policy Implications
Positivist	What is the impact of user fees on service utilization and across different groups of patients?	Levels at which user fees should be set.Which population groups should be exempted from fees.
Critical realist and relativist	Why were user fees introduced and how was equity conceived?	Strengthening the voice of the poor in policy and implementation processes so as to promote more pro-poor policies.
	How do out-of-pocket payments interact with other influences on care seeking?	Should policy focus on addressing user fees as the key obstacle to utilization, or would it also be necessary (or perhaps even more important) to address other barriers to care seeking.
	How is user fee policy experienced by those implementing it?	Strategies to empower health staff in the policy development and implementation processes, so as to ensure that the framing of the policy takes account of their concerns, as a means to strengthening implementation.

## Building Capacity for the Field

So, given these problems, what can be done to take advantage of the growing momentum to develop capacities in the field?

**Figure 1:**
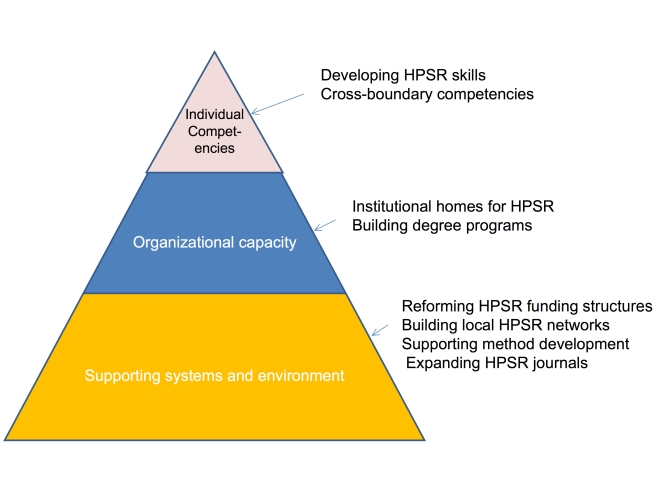
Dimensions of capacity in the HPSR field.

Approaches to capacity development often conceive of capacity as a hierarchy starting from broad systems and structural issues and working up to specific individual skills and competencies. We use a similar approach to describe what needs to be done in the HPSR field (Figure 1).

### Supporting Systems and the Research Environment

HPSR needs to be driven by local actors who have an intimate understanding of their own health systems and the challenges that they face. Reforming systems for funding HPSR appears crucial in this respect. Despite repeated international commitments to increasing domestic resources for health research, in most LMICs extremely limited national resources are applied to HPSR. Increases in domestic commitments are important, although likely to be difficult to achieve without stronger policy-maker demand for HPSR. In addition, measures that pool a proportion of international research resources at the country level and develop local systems and capacities to allocate these resources in an informed manner need to be explored. The widespread use of consultants could be addressed through dedicating a proportion of consultancy funding to local research departments and organizations as core funding, thus both reducing the temptation for researchers to chase short-term projects, and enabling greater responsiveness to local policy-makers than project funding allows.

The emphasis on international funding and research consortia means that networks between researchers within a country are often neglected. Strengthening national research networks can help achieve multiple goals: strengthening the focus on national research priorities, enhancing capacity through bringing together researchers with differing disciplinary skills, and facilitating longer-term trust-based networks. Developing closer ties between health care providers and managers, policy-makers, and health systems researchers can help promote an organizational culture of evidence-informed decision-making across the health sector, facilitate access to research sites for researchers, and generate a stronger focus on locally identified research questions.

Greater shared understanding of the theoretical frames and methodological approaches that drive the field is also needed. HPSR journals with a focus on LMICs publish primarily empirical papers. Greater opportunities for conceptual work and methodological dialogue are needed, including journal papers, online resources and exchange, and methodological tracks within HPSR conferences. The work of the United States-based Health Services Research Methods Council of AcademyHealth provides one possible approach, providing online and in person methods seminars, and a library of online research resources among other things (see http://www.academyhealth.org/Training/ResourceDetail.cfm?ItemNumber=2418). The Alliance for Health Policy and Systems Research, as the only multilateral agency with a mandate focused on HPSR, has a natural leadership position in supporting such activities, although this should be done in partnership with others. Comparing the evolution of health services research in high-income countries with HPSR in LMICs may also be instructive—while the two fields have developed in parallel, there are surely shared lessons.

Relatively few journals focus on publishing HPSR relevant to LMICs, and there are very few regional or country-specific journals of this nature. As a consequence, researchers try to publish in journals that do not understand the field well—whether social science disciplinary journals or broader public health or biomedical journals—and may receive poorly informed reviews, or have papers rejected outright for either being “too applied” or not applying the kind of research methods that are recognized by the biomedical sciences. Some papers of considerable national or regional policy interest remain unpublished as they do not fit well with priorities of international journals. Expectations regarding paper length and structure also limit HPSR publishing opportunities. Biomedical journals typically expect short papers (3,000–3,500 words in length) with findings presented in tabular formats; for qualitative and mixed-methods studies this is inappropriate. Reduced to short papers for publication, HPSR studies may lose richness and nuance. Mixed-method studies may be split into qualitative and quantitative components and published separately with consequent loss of the synergies between these approaches.

The lack of appropriate outlets for publication creates indirect problems too. Difficulties in publishing may undermine the career development of HPS researchers, and the lack of country and regional journals reinforces the financial incentives for researchers to focus on global HPSR priorities rather than national ones. New HPSR journals, particularly those focused on specific countries or regions, are needed, and general health journals should review their policies in terms of reviewer identification and article length if they seriously wish to accommodate HPSR papers.

### Organizational Capacity

HPSR often occupies a tenuous home within universities and academic departments. While much HPSR takes place within schools of public health, few schools have departments dedicated to health policy and systems; instead, HPSR may be housed in departments of epidemiology, community/public health, or health management. Alternatively, HPS researchers may be located in discipline-specific departments (such as economics departments) that may not fully appreciate the applied or inter-disciplinary nature of the field. In such contexts, HPS researchers may struggle for due recognition of their work and be pressured to adopt particular types of research paradigms and methods. For HPSR to develop as a field, dedicated and supportive homes within universities are required and research leadership may need to be educated about the inter-disciplinary nature and contemporary relevance of HPSR. Positioning HPSR as being at the cutting edge of current efforts to work across the boundaries of social and biological sciences [1] may help stimulate enthusiasm and support.

Given the policy-relevant and question-focused nature of HPSR, it is also important to support think tanks and other types of research institutes that can provide stable institutional environments for HPSR as well as offer opportunities for close engagement with policy processes. Case studies of such institutes have highlighted the importance of secure funding, such as endowments, as well as strong links to policy-makers in the success of these institutes [16].

Training curricula for HPSR are lacking and relatively few courses teach HPSR methods relevant to LMICs; instead, HPSR is typically a minor component of the material covered. To generate greater shared understanding of methods, HPSR curricula need to be developed that promote a greater degree of shared perspectives, methodological understandings, and language among those who work in the field. A common approach to categorizing, organizing, and teaching the multiple theoretical frames and methods for HPSR should be developed, as should guidance to help researchers select which type of approach will work best for different types of HPSR questions. Training curricula need to provide a solid orientation to the paradigmatic differences described in the second paper of this series. Two recently funded European Union projects are addressing these issues (CHEPSAA the Consortium for Health Policy and Systems Analysis in Africa and ARCADE, the African Regional Capacity Development for Health Services and Systems Research grant), but the discussions ultimately need to engage the whole of the HPSR community.

### Individual Competencies

People come to HPSR with varied backgrounds and needs: some may be trained social scientists who have little understanding of the health sector but wish to apply their skills to health systems questions. Many come from broad public health backgrounds, perhaps with experience in disease control programs. Others are clinical practitioners or researchers who usually have very limited exposure to social sciences. Given the diversity of individuals entering the field, training programs need to be tailored to the needs of different types of entrants, while still ensuring a common basic training in HPSR concepts, approaches, and terminology.

For the inter-disciplinary health systems researcher, a post-graduate training in HPSR is desirable. Much support to research capacity development for HPSR to date has taken the form of short course training [4]. Short courses appear to scale up capacity rapidly to conduct HPSR, but their utility in producing rounded, inter-disciplinary health systems researchers is doubtful. It would be better to invest in graduate training programs and scholarship funding, as student support remains a problem for many HPSR academic programs. Short courses may have a limited role to play in orienting social scientists coming from different fields to HPSR, keeping qualified researchers up-to-date in new developments in the field, or in helping policy- and other decision-makers gain an appreciation of the HPSR field.

The skills needed to be effective in inter-disciplinary research are often compared to cultural competencies: the ability to listen to other points of view, respect different positions, and communicate ideas effectively without resort to jargon and disciplinary-specific terminology [17]. Other competencies that support work across boundaries include an ability to see the big picture and a strong ethical orientation [18]. HPS researchers need to be consummate boundary crossers, traversing disciplines, organizations, and professions. Training programs should reinforce such competencies, encouraging diverse students to work together. Innovative approaches to structuring academic programs can also promote work across traditional boundaries through interspersing didactic training with practicums or internships, and promoting interaction with practitioners.

Particularly in light of the hostile organizational environments in which some HPS researchers work, mentorship may be key to sustaining interest and skill development. Very few mentorship programs exist and their development requires careful planning to motivate and support overstretched senior researchers who could act as mentors. Given the challenges in developing mentorship schemes, efforts to build communities of practice among HPS researchers within countries and at regional levels may also be helpful.

## Conclusions

HPSR is currently at a tipping point. As a new scientific field develops there are likely to be struggles over ideas, resources, and paradigmatic dominance [19]. As HPS researchers we have all spent time repeatedly explaining and justifying what we do, and why we use the methods we do. In its emergent phase HPSR has been characterized by fluidity and sometimes a lack of clarity: the first paper in this series described how HPSR has emerged from questions bubbling up from the field [9].

With increased recognition of the important role that HPSR needs to play in achieving health goals there is a healthy intensification of questions regarding the nature of the field. Given the rush of new blood and interest in HPSR, we urgently need to move beyond individual explanations of what we do. Instead, we need to take advantage of the current interest to develop the programs and structures of a fully-fledged scientific field with core curricula, text books, scientific meetings, communities of practice, academic departments, and journals. Through investment in the range of activities described in this paper (see also Box 1), HPSR can develop into a more crystalline form, underpinned by shared and inter-disciplinary understandings. Only with this consolidated intellectual development can the field of HPSR realize its full potential to contribute new knowledge for health systems strengthening.

**Box 1. An Agenda for Action**

- Reform systems for funding HPSR so as to create stronger national ownership
- Build capacity and understanding of HPSR to enable country level policy- and decision-makers and managers to effectively “manage” the ownership
- Develop stronger HPSR networks at the national and sub-national levels
- Build opportunities for methodological dialogue and exchange
- Develop more HPSR journals, especially at country and regional levels
- Identify and build sustainable institutions for HPSR in universities and in independent research institutes
- Focus training on post-graduate courses and develop core training curricula for HPSR
- Build inter-disciplinary competencies among HPS researchers

## Abbreviations

HPSR: Health Policy and Systems Research
LMIC: low- and middle-income country
